# Supplementary material for: A new program for systematically enhancing cognitive reserve in healthy adults: A pilot randomized active-controlled clinical trial
Source: PLoS One. 2025 Oct 1;20(10):e0331193. doi: 10.1371/journal.pone.0331193 (PMC12488004; doi:10.1371/journal.pone.0331193)
Supplement: S1 File — This file includes the original study protocol and the statistical plan that outlines the study objectives, methodology and prespecified statistical methods. (PDF) [file pone.0331193.s001.pdf]

MENTAL TRAINING TECH 24.5
STUDY PROTOCOL AND STATISTICAL ANALYSIS PLAN

Protocol Scientific Tittle:	Evaluation of the Effectiveness of the MTT24.5 Cognitive Training Program Based on Neuroplasticity Stimulation on Cognitive Skills in Adults from the General Population
Protocol Version:	3.0/final
Principal Investigator:	Carol Kotliar
Protocol design:	Clinical randomized open trial, active-controlled.

Protocol and SAP Signatures

I give my approval for the SAP entitled "MENTAL TRAINING TECH 24.5 STUDY PROTOCOL AND STATISTICAL ANALYSIS PLAN"

version 3.0, dated 21/01/2023.

Principal Investigator

Name: Carol Kotliar

Signature: CKotliar

Date: 25/01/2023

Table of Contents

1. Administrative information ***3***
1.1. Document History ***3***
1.2. List of Abbreviations and Definitions of Terms ***3***
2. Introduction ***4***
3. Protocol summary ***4***
3.1 Background ***4***
3.2 Study hypothesis ***4***
3.3 Study objectives ***5***
3.4 Study design ***5***
3.5 Interventions description ***5***
3.6 Population ***6***
3.7 Sample size ***6***
3.8 Data collection ***6***
3.9 Study outcomes ***7***
4. Outcomes definitions ***7***
4.1 Primary outcome ***7***
4.2 Secondary outcomes ***7***
5. Statistical analyses ***8***
5.1 Subject Disposition ***8***
5.2 Distribution of socio demographic and clinical characteristics ***8***
5.3 Statistical analysis methods ***8***
5.3.1 Objective 1 ..... ***8***
5.3.2 Objective 2 ..... ***9***
5.4 Handling of Missing, Unused, and Spurious Data ***10***
5.5 Confidence intervals and p-values ***10***
5.6 Statistical software employed ***10***
5.7 Reporting conventions ***10***
6. Dummy tables ***11***
Figure 1: Consort ***11***
..... ***11***
Table 1. Socio-demographic characteristics by group ***12***
Table 2. Effect of MTT 24.5 program on cognitive abilities outcomes ***13***
7.References ***14***

1. Administrative information

1.1. Document History

Version	Date of Issue	Summary of Change
1.0	03/01/2023	Initial version
2.0	07/01/2023	Revised version<br>• 3.3 Primary objective #1: The 6 score dimensions have been specified.<br>• 3.3 Secondary objective: Since information regarding tolerance and adherence is available, assessment of this objective will be conducted.<br>• 3.5 The definition of the MTT24.5 program has been updated.<br>• 3.7 Power calculation has been incorporated.<br>• 4.2 Outcomes related to adherence have been defined.<br>• 5.3.4 Statistical method for adherence outcome analysis has been outlined.
3.0	21/01/2023	Final version

1.2. List of Abbreviations and Definitions of Terms

ACE	Addenbrooke's Cognitive Examination
CRS	Cognitive Reserve Score
IQR	Inter quartile range
MTT24.5	Mental Training Tech 24.5
SCD	Subjective cognitive decline
SD	Standard deviation
TECH	High-impact cognitive stimulation modalities
AAS	Acetylsalicylic Acid

2. Introduction

The purpose of this document is to describe the protocol and planned analysis and reporting for the neuroplasticity applied program “MENTAL TRAINING TECH 24.5”

3. Protocol summary

3.1 Background

The human brain can change and adapt throughout life, challenging the previous belief that the process of brain aging was irreversible. This paradigm shift, driven by scientific discoveries such as those of Eric Kandel, highlights the importance of neuroplasticity, the brain's ability to reorganize, in preserving and recovering cognitive functions. Therefore, it is necessary to incorporate brain training programs into culture and medicine to harness this adaptive capacity of the brain and improve quality of life at all stages.

Based on this knowledge situation, the applied neuroplasticity program "Mental Training Tech 24.5" (MTT24.5) has been developed as a method for inducing brain plasticity to enhance, protect, and preserve cognitive abilities, fostering from a physiological model of brain connection growth and strengthening. The increase in life expectancy has triggered the challenge of finding options to reduce age-related cognitive decline. In this context, the explicit stimulation of new learning through neuroplasticity offers an opportunity to enhance cognitive reserve as a surrogate marker, with the expectation that this change may delay the threshold for the decline of cognitive functions such as memory, analytical reasoning, and verbal fluency. Enhanced cognitive reserve, along with the neuroplastic activation of previously inactive areas in the adult brain, has been linked to a reduction in the manifestations of dementia. Therefore, cognitive training for adults emerges as a promising response to the challenge of longevity. The present protocol is considered an initial pilot study to validate the effectiveness of the new cognitive stimulation method, MTT24.5, and will serve as the foundation for future research exploring changes in brain activation and structure associated with this method

3.2 Study hypothesis

- *Hypothesis 1*

  The MTT24.5 cognitive training program is associated with cognitive brain functional changes, as it may increase performance in memory and/or attention and/or verbal fluency and/or language and/or visuospatial skills in adults without clinical cognitive impairment or with subjective cognitive decline (SCD).

- *Hypothesis 2*

Different baseline conditions may modulate responses to the cognitive program, including a) baseline cognitive reserve, b) history of dementia, c) non-communicable chronic diseases, d) lifestyle habits, and e) medication use.

3.3 Study objectives

- ***Primary objectives***

1. Evaluate the effects of MTT24.5 on cognitive abilities such as attention, memory, verbal fluency, and visuospatial skills.
2. Determine if there are response phenotypes based on the distribution of individual baseline characteristics, such as age, gender, lifestyle factors, medical history, medications, family history of dementia, diet, and baseline cognitive reserve score.

- ***Secondary objective***

Analyze the tolerance and adherence to MTT24.5, and its association with outcomes regarding cognitive variables.

3.4 Study design

It is a prospective, open randomized controlled clinical study that evaluates the effectiveness and tolerance of a cognitive intervention.

3.5 Interventions description

- ***Control Group***

Participants in this group will not receive any specific cognitive training program during the study period. They will continue with their usual activities and routines.

- ***Intervention Group:***

Participants in this group will receive the cognitive training program MTT24.5.

The MTT24.5, developed as a binomial DATA (or KNOWLEDGE) + TECH, provides the brain with new knowledge (DATA) classified in formal, natural (biological), social, and cultural areas. The TECH consists of 100 high-impact cognitive stimulation modalities designed to enhance memory, attention, verbal fluency, and visuospatial skills. During the program, the brain receives 40 new pieces of knowledge, and the 100 TECHS work to integrate them into the participant's daily life. This integration intends to promote changes in brain functionality, improves synaptic efficiency, and achieves permanent plastic changes. The program's duration is approximately 24.5 hours spread across 12 weeks including in-person training classes of 1.5 hours each week.

3.6 Population

- ***Inclusion criteria***
  - Adults aged 21 years and above.
  - Ability to understand the instructions for the training tasks.
- ***Exclusion criteria:***
  - Planned absence that would hinder participation in the program.
  - Hearing, visual, or motor deficits that would impede participation in the program.
  - History of severe degenerative neurological diseases as these conditions may significantly impact brain plasticity and cognitive abilities.
  - History of severe psychiatric disorders as they may influence brain reorganization and complicate interpretation of the results.
  - Unstable treatment or planned changes in medication that may affect brain function and alter results.
  - History of current or recent excessive substance use (within the past 6 months) including alcohol or drugs as these substances may influence brain function and neuroplasticity.

3.7 Sample size

The sample size of 76 subjects (56 cases and 20 controls) will provide a power of at least 80% to detect a minimum difference of 4 points in the score improvement between the two groups, assuming a maximum standard deviation of 5.4 points. This minimum difference of 4 points corresponds to a minimum effect size of 0.8.

3.8 Data collection

Information will be collected on study specific forms/procedure.

- ***Cognitive Reserve Score (CRS):*** It consists of a validated cognitive reserve scale used with authorization from Roldan L. et al. The CRS records the frequency of cognitively stimulating activities carried out throughout life. A total of 24 items are distributed across four aspects: activities of daily living, education/information, hobbies, and social life. The CRS will be obtained for each subject at baseline (pre intervention).
- ***Medical and Lifestyle History Form***. Self-administered medical and lifestyle history form called STEPS 5, adapted from www.who.int/chp/steps, and previously used in the Latin American OPTIMO study. This form will be administrated for each subject at baseline (pre intervention).

- ***The Addenbrooke's Cognitive Examination-III.*** Brief cognitive test that evaluates five cognitive abilities: attention, memory, verbal fluency, language, and visuospatial skills. The ACE score will be obtained for each subject at baseline (pre intervention) and post intervention.

3.9 Study outcomes

- ***Primary outcome***
  - Cognitive ability improvement
- ***Secondary outcomes – related to cognitive abilities***
  - Memory ability improvement
  - Attention ability improvement
  - Orientation ability improvement
  - Verbal fluency ability improvement
  - Language ability improvement
  - Visuospatial skills ability improvement

4. Outcomes definitions

4.1 Primary outcome

The primary study outcome, cognitive ability improvement, will be assessed through the difference between the post intervention ACE score and the pre intervention ACE score. This score ranges from 0 to 100.

4.2 Secondary outcomes

- ***Outcomes related to cognitive abilities***

  The ACE score comprises six dimensions, each evaluating a different domain: Memory, attention, orientation, verbal fluency, language, and visuospatial skills abilities.

  Each ability outcome improvement will be assessed using the respective sub-dimensions of the ACE score and computing the difference between post intervention and pre intervention sub-score:

  - Memory ability sub-score improvement
  - Attention ability sub-score improvement
  - Orientation ability sub-score improvement
  - Verbal fluency ability sub-score improvement

- Language ability sub-score improvement
- Visuospatial skills ability sub-score improvement

The memory ability sub-score ranges from 0 to 26, the attention abilities sub-score ranges from 0 to 8, the attention abilities sub-score ranges from 0 to 10, the verbal fluency sub-score ranges from 0 to 14, the language sub-score ranges from 0 to 16, and the visuospatial skills abilities sub-score ranges from 0 to 26.

- ***Outcomes related to adherence:***

Compliance with the study protocol is assessed via:

(1) the number of days that participant completed ("on site days")

5. Statistical analyses

5.1 Subject Disposition

A CONSORT flow diagram will be used to summarize the number of subjects who were:

- Enrolled
- Enrolled to each group
- Discontinued in each group
- Included in the Cognitive analysis in each group

5.2 Distribution of socio demographic and clinical characteristics

All continuous variables will be summarized using the following descriptive statistics: mean, standard deviation (SD) and range if data presented a normal distribution and median, inter quartile range (IQR) and range if data skewed. The frequency and percentages (based on the non-missing sample size) of observed levels will be reported for all categorical measures.

5.3 Statistical analysis methods

5.3.1 Objective 1

The mean baseline ACE score and standard deviation (SD) will be reported for both the intervention and control groups. Additionally, the improvement from baseline to the end of the program will also be presented for each group.

To assess the impact of MTT24.5 on cognitive abilities, a linear model will be utilized for each cognitive domain (global, memory, attention and orientation, verbal fluency, language, and visuospatial skills). Each model will use the improvement score value as the outcome variable

and the group (intervention and control) as the independent variable. The group term will evaluate the non-standardized intervention effect, indicating whether the mean improvement in scores (change from baseline) differs between the control and intervention groups. The effect size is defined as the difference between the mean improvement on the intervention group and the mean improvement on the control group.

To compute standardized effect sizes, a linear model will be utilized, incorporating the scaled outcome for each domain. The scaled outcome will be computed using the formula: (improvement score - mean (improvement score)/ SD(improvement score). This transformation facilitates comparisons across different scores.

The fulfillment of model assumptions will be assessed, and if they are not met, proposals for improvement will be suggested.

5.3.2 Objective 2

To identify potential response phenotypes based on individual baseline characteristics (including age, gender, medical history, medications, family history of dementia, and baseline cognitive reserve score), a linear model will be employed for each covariate of interest. The primary outcome will be modelled with the covariate of interest, the group variable, and their interaction. This interaction term will assess whether the intervention effect varies across covariate groups.

To summarize the results, a forest plot displaying the standardized effect size for each covariate group will be generated.

Covariate / Subgroups of interest:

- Sex (female, male)
- Age (<=65 years, >65 years)
- Years of Education (<=12 years, >12 years)
- Cognitive reserve (low, high)
- ACE score at baseline (<85, >85)
- Diabetes history (yes, no)
- Hypertension history (yes, no)
- Dyslipidemia history (yes, no)
- Dementia family history (yes, no)
- Statins /AAS use (yes, no)

5.4 Handling of Missing, Unused, and Spurious Data

Variables with more than 20% of missing data will not be included in the analysis. For variables that have less than 20% of missing values, firstly it will be investigated and tried to complete the missing data. Available data will be included in the data listings and tabulations showing for each report the actual total. No imputation techniques will be used for missing data.

5.5 Confidence intervals and p-values

All applicable statistical tests will be 2-sided and will be performed using a 5% significance level. All confidence intervals presented will be 95% and two-sided.

5.6 Statistical software employed

The statistical software R version 4.3.0 will be used for the analyses.

5.7 Reporting conventions

P-values ≥0.001 will be reported to 3 decimal places; p-values less than 0.001 will be reported as “<0.001”. The mean, standard deviation, and any other statistics other than quantiles, will be reported to one decimal place greater than the original data. Quantiles, such as median, or minimum and maximum will use the same number of decimal places as the original data. Estimated parameters, not on the same scale as raw observations will be reported to 3 significant figures.

6. Dummy tables

Figure 1: Consort

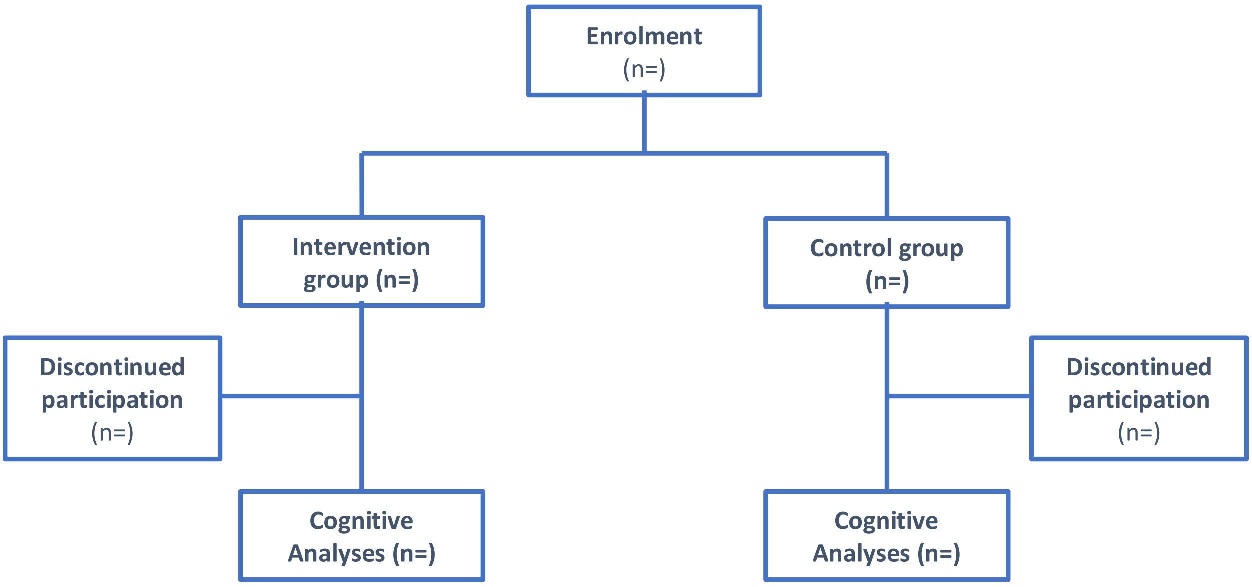

Table 1. Socio-demographic characteristics by group

	Control group	Intervention Group
	**n/N (%)**	**n/N (%)**
**Socio-demographic characteristics**		
**Sex**		
Male		
Female		
**Age***		
**Age categorized**		
<=65 years		
>65 years		
**Years of education***		
**Mediterranean diet**	-	
**Physical activity**	-	
**Clinical characteristics**		
**Cognitive reserve**		
Low		
High		
**Diabetes history**		
**Hypertension history**		
**Dyslipidemia history**		
**Myocardial infarction**		
**Previous cerebrovascular accident**		
**Sleep apnea syndrome**		
**Dementia family history**		

*Mean and (SD) will be reported

Table 2. Effect of MTT 24.5 program on cognitive abilities outcomes

	Control group (N=)	Intervention Group (N=)	P-value
**Primary Outcome**			
Global cognitive ability			
Score at baseline, mean (±SD)			
Improvement from baseline, mean (±SD)			
Non-standardized effect size (95% CI)*			
Standardized effect size (95% CI)**			
**Secondary Outcomes**			
Attention cognitive ability			
Score at baseline, mean (±SD)			
Improvement from baseline, mean (±SD)			
Non-standardized effect size (95% CI)*			
Standardized effect size (95% CI)**			
Orientation cognitive ability			
Score at baseline, mean (±SD)			
Improvement from baseline, mean (±SD)			
Non-standardized effect size (95% CI)*			
Standardized effect size (95% CI)**			
Memory cognitive ability			
Score at baseline, mean (±SD)			
Improvement from baseline, mean (±SD)			
Non-standardized effect size (95% CI)*			
Standardized effect size (95% CI)**			
Verbal fluency cognitive ability			
Score at baseline, mean (±SD)			
Improvement from baseline, mean (±SD)			
Non-standardized effect size (95% CI)*			
Standardized effect size (95% CI)**			
Language cognitive ability			
Score at baseline, mean (±SD)			
Improvement from baseline, mean (±SD)			
Non-standardized effect size (95% CI)*			
Standardized effect size (95% CI)**			
Visuospatial skills cognitive ability			
Score at baseline, mean (±SD)			
Improvement from baseline, mean (±SD)			
Non-standardized effect size (95% CI)*			
Standardized effect size (95% CI)**			

Abbreviations: CI, confidence interval; SD, standard deviation
**Non-standardized effect size defined as the difference between the mean improvement on the intervention group and the mean improvement on the control group
**The standardized effect size defined as the difference between the mean of the scaled improvement on the intervention group and the mean of the scaled improvement on the control group

Figure 2. Effect of MTT 24.5 program on cognitive abilities by covariates groups

Forest plot

7. References

1. Ball K, Berch DB, Helmers KF, et al. Effects of Cognitive Training Interventions With Older Adults: A Randomized Controlled Trial. *JAMA.* 2002;288(18):2271–2281. doi:10.1001/jama.288.18.2271
2. Belleville S, Mellah S, Boller B, Ouellet É. Activation changes induced by cognitive training are consistent with improved cognitive reserve in older adults with subjective cognitive decline. Neurobiol Aging. 2023 Jan;121:107-118. doi: 10.1016/j.neurobiolaging.2022.10.010. Epub 2022 Oct 23. PMID: 36401900.
3. Megan A. Jennings, Robert A. Cribbie. Comparing Pre-Post Change Across Groups: Guidelines for Choosing between Difference Scores, ANCOVA, and Residual Change Scores, J. data sci. 14(2022), no. 2, 205-230, DOI 10.6339/JDS.201604_14(2).0002
4. Rebok GW, Ball K, Guey LT, Jones RN, Kim HY, King JW, Marsiske M, Morris JN, Tennstedt SL, Unverzagt FW, Willis SL; ACTIVE Study Group. Ten-year effects of the advanced cognitive training for independent and vital elderly cognitive training trial on cognition and everyday functioning in older adults. J Am Geriatr Soc. 2014 Jan;62(1):16-24. doi: 10.1111/jgs.12607. Epub 2014 Jan 13. PMID: 24417410; PMCID: PMC4055506.
5. Hardy JL, Nelson RA, Thomason ME, Sternberg DA, Katovich K, Farzin F, et al. (2015) Enhancing Cognitive Abilities with Comprehensive Training: A Large, Online, Randomized, Active-Controlled Trial. PLoS ONE 10(9): e0134467. doi:10.1371/journal.pone.0134467
6. Scarmeas N, Stern Y. Cognitive reserve and lifestyle. *J Clin Exp Neuropsychol*. 2003;25(5):625 633.
7. May A. experience-dependent structural plasticity in the adult human brain*. Trends Cogn Sci.* 2011; 15:475-82. doi: 10.1016/j. tics.2011.08.002
