# Supplementary material for: A new program for systematically enhancing cognitive reserve in healthy adults: A pilot randomized active-controlled clinical trial
Source: PLoS One. 2025 Oct 1;20(10):e0331193. doi: 10.1371/journal.pone.0331193 (PMC12488004; doi:10.1371/journal.pone.0331193)
Supplement: S2 File — This file contains the original version of the study protocol in Spanish, as approved by the Institutional Review Board (IRB) prior to study initiation. (PDF) [file pone.0331193.s002.pdf]

PROTOCOLO DE ESTUDIO Y PLAN DE ANÁLISIS ESTADÍSTICO MENTAL TRAINING TECH 24.5

Título Científico del Protocolo: Evaluación de la Efectividad del Programa de Entrenamiento Cognitivo MTT24.5 Basado en la Estimulación de Neuroplasticidad sobre las Habilidades Cognitivas en Adultos de la Población General.

Versión del Protocolo: 3.0/final

Investigadora Principal: Carol Kotliar

Diseño del Protocolo: Estudio clínico aleatorizado abierto, controlado con tratamiento activo.

Firmas del Protocolo y del SAP

Otorgo mi aprobación al SAP titulado "MENTAL TRAINING TECH 24.5 ESTUDIO PROTOCOLO Y PLAN DE ANÁLISIS ESTADÍSTICO", versión 3.0, fechado el 21/01/2023.

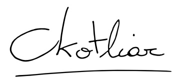

Investigadora Principal
Nombre: Carol Kotliar
Firma:
Fecha: 25/01/2023

Tabla de Contenidos

1. Información Administrativa 3
   1.1. Historia del Documento 3
   1.2. Lista de Abreviaturas y Definiciones de Términos 3
2. Introducción 3
3. Resumen del Protocolo 3
   3.1. Antecedentes 3
   3.2. Hipótesis del Estudio 4
   3.3. Objetivos del Estudio 4
   3.4. Diseño del Estudio 4
   3.5. Descripción de las Intervenciones 5
   3.6. Población 5
   3.7. Tamaño de la Muestra 5
   3.8. Recopilación de Datos 6
   3.9. Resultados del Estudio 6
4. Definiciones de los Resultados 6
   4.1. Resultado Primario 6
   4.2. Resultados Secundarios 6
5. Análisis Estadísticos 7
   5.1. Disposición de los Sujetos 7
   5.2. Distribución de las Características Socio-Demográficas y Clínicas 7
   5.3. Métodos de Análisis Estadísticos 7
   5.3.1. Objetivo 1 7
   5.3.2. Objetivo 2 8
   5.4. Manejo de Datos Perdidos, No Usados y Espurios 8
   5.5. Intervalos de Confianza y Valores p 9
   5.6. Software Estadístico Empleado 9
   5.7. Convenciones de Reporte 9
6. Tablas de Referencia 9
   Figura 1: Diagrama CONSORT 9
   Tabla 1. Características Socio-Demográficas por Grupo 10
   Tabla 2. Efecto del Programa MTT 24.5 sobre los Resultados de las Habilidades Cognitivas 10
7. Referencias 11

1. Información Administrativa

1.1. Historia del Documento

Versión	Fecha de Emisión	Resumen de Cambios
1.0	03/01/2023	Versión inicial
2.0	07/01/2023	Versión revisada
3.0	21/01/2023	Versión final

1.2. Lista de Abreviaturas y Definiciones de Términos

- **ACE**: Examen Cognitivo Addenbrooke
- **CRS**: Puntaje de Reserva Cognitiva
- **IQR**: Rango Intercuartil
- **MTT24.5**: Mental Training Tech 24.5
- **SCD**: Declive Cognitivo Subjetivo
- **SD**: Desviación estándar
- **TECH**: Modalidades de estimulación cognitiva de alto impacto
- **AAS**: Ácido Acetilsalicílico

---

2. Introducción al estudio

El propósito de este documento es describir el protocolo y el análisis y reporte planificados para el programa de neuroplasticidad aplicada “MENTAL TRAINING TECH 24.5” (MTT24.5).

---

3. Resumen del Protocolo

3.1. Antecedentes

El cerebro humano tiene la capacidad de cambiar y adaptarse a lo largo de la vida, lo que desafía la creencia anterior de que el envejecimiento cerebral era irreversible. Este cambio de paradigma, impulsado por descubrimientos científicos como los de Eric Kandel, destaca la importancia de la neuroplasticidad, la capacidad del cerebro para reorganizarse, en la preservación y recuperación de las funciones cognitivas. Por lo tanto, es necesario incorporar programas de entrenamiento cerebral en la cultura y la medicina para aprovechar esta capacidad adaptativa del cerebro y mejorar la calidad de vida en todas las etapas.

En este contexto, el programa de neuroplasticidad aplicada "Mental Training Tech 24.5" (MTT24.5) ha sido desarrollado como un método para inducir plasticidad cerebral que mejore, proteja y preserve las habilidades cognitivas, basándose en un modelo fisiológico

de crecimiento y fortalecimiento de las conexiones cerebrales. El aumento de la esperanza de vida ha generado el desafío de encontrar opciones para reducir el declive cognitivo relacionado con la edad. En este sentido, la estimulación explícita de nuevos aprendizajes a través de la neuroplasticidad ofrece una oportunidad para mejorar la reserva cognitiva, con la esperanza de que este cambio pueda retrasar el umbral de declive de funciones cognitivas como la memoria, el razonamiento analítico y la fluidez verbal. Una mayor reserva cognitiva, junto con la activación neuroplástica de áreas previamente inactivas del cerebro adulto, se ha vinculado a una reducción en las manifestaciones de la demencia. Por lo tanto, el entrenamiento cognitivo para adultos se presenta como una respuesta prometedora al desafío de la longevidad. El presente protocolo se considera un estudio piloto inicial para validar la efectividad del nuevo método de estimulación cognitiva MTT24.5 y servirá como base para futuras investigaciones que exploren los cambios en la activación y la estructura cerebral asociados con este método.

3.2. Hipótesis del Estudio

- **Hipótesis 1**: El programa de entrenamiento cognitivo MTT24.5 está asociado con cambios funcionales cerebrales cognitivos, ya que puede incrementar el rendimiento en memoria y/o atención y/o fluidez verbal y/o lenguaje y/o habilidades visoespaciales en adultos sin deterioro cognitivo clínico o con declive cognitivo subjetivo (SCD).
- **Hipótesis 2**: Diferentes condiciones basales pueden modular las respuestas al programa cognitivo, incluyendo a) reserva cognitiva basal, b) historia de demencia, c) enfermedades crónicas no transmisibles, d) hábitos de vida, y e) uso de medicamentos.

3.3. Objetivos del Estudio

- **Objetivos Primarios**
  1. Evaluar los efectos de MTT24.5 sobre las habilidades cognitivas, como atención, memoria, fluidez verbal y habilidades visoespaciales.
  2. Determinar si existen fenotipos de respuesta basados en la distribución de características individuales basales, tales como edad, sexo, factores de estilo de vida, historial médico, medicamentos, historia familiar de demencia, dieta y puntaje de reserva cognitiva basal.
- **Objetivo Secundario** Analizar la tolerancia y la adherencia a MTT24.5, y su asociación con los resultados en variables cognitivas.

3.4. Diseño del Estudio

Es un estudio prospectivo, randomizado y controlado, abierto, que evalúa la efectividad y tolerancia de una intervención cognitiva.

3.5. Descripción de las Intervenciones

- **Grupo Control**
  Los participantes en este grupo no recibirán ningún programa específico de

entrenamiento cognitivo durante el período del estudio. Continuarán con sus actividades y rutinas habituales.

- **Grupo de Intervención**
  Los participantes en este grupo recibirán el programa de entrenamiento cognitivo MTT24.5.
  El MTT24.5, desarrollado como un binomio de DATOS (o CONOCIMIENTOS) + TECH, proporciona al cerebro nuevos conocimientos (DATOS) clasificados en áreas de las ciencias formales, naturales (biológica), sociales y culturales. Las TECHS consisten en 100 modalidades de estimulación cognitiva de alto impacto diseñadas para mejorar la memoria, la atención, la fluidez verbal y las habilidades visoespaciales. Durante el programa, el cerebro recibe 40 piezas nuevas de conocimiento, y las 100 TECHS trabajan para integrarlas en la vida diaria del participante. Esta integración tiene como objetivo promover cambios en la funcionalidad cerebral, mejorar la eficiencia sináptica y lograr cambios plásticos permanentes. La duración del programa es de aproximadamente 24.5 horas distribuidas en 12 semanas, que incluyen clases de entrenamiento presencial de 1.5 horas cada semana.

3.6. Población

- **Criterios de Inclusión**
  - Adultos de 21 años o más.
  - Capacidad para comprender y realizar las instrucciones de las tareas de entrenamiento.
  - Firma de informe de consentimiento
- **Criterios de Exclusión**
  - Ausencia planificada que dificultaría la participación en el programa.
  - Déficits auditivos, visuales o motores que impidan la participación en el programa.
  - Historia de enfermedades neurológicas degenerativas graves, ya que estas condiciones pueden afectar significativamente la plasticidad cerebral y las habilidades cognitivas.
  - Historia de trastornos psiquiátricos graves, ya que estos pueden influir en la reorganización cerebral y complicar la interpretación de los resultados.
  - Tratamiento inestable o cambios planificados en la medicación que puedan afectar la función cerebral y alterar los resultados.
  - Historia de consumo excesivo de sustancias actuales o recientes (en los últimos 6 meses), incluidas alcohol o drogas, ya que estas sustancias pueden influir en la función cerebral y la neuroplasticidad.

3.7. Tamaño de la Muestra

El tamaño de la muestra de 76 sujetos (56 casos y 20 controles) proporcionará una potencia de al menos el 80% para detectar una diferencia mínima de 4 puntos en la mejora del puntaje entre los dos grupos, asumiendo una desviación estándar máxima de 5.4 puntos. Esta diferencia mínima de 4 puntos corresponde a un tamaño de efecto mínimo de 0.8.

3.8. Recopilación de Datos

La información se recopilará mediante formularios/procedimientos específicos del estudio.

- **Puntaje de Reserva Cognitiva (CRS)**: Consiste en una escala validada de reserva cognitiva utilizada con la autorización de Roldán L. et al. El CRS registra la frecuencia de actividades cognitivamente estimulantes realizadas a lo largo de la vida. Un total de 24 ítems están distribuidos en cuatro aspectos: actividades de la vida diaria, educación/información, pasatiempos y vida social. El CRS se obtendrá para cada sujeto en el inicio del estudio (antes de la intervención).
- **Formulario de Historia Médica y de Estilo de Vida**: Formulario autoadministrado de historia médica y de estilo de vida denominado STEPS 5, adaptado de www.who.int/chp/steps, y previamente utilizado en el estudio latinoamericano OPTIMO. Este formulario será administrado a cada sujeto al inicio del estudio (antes de la intervención).
- **Examen Cognitivo Addenbrooke-III**: Prueba cognitiva que evalúa cinco habilidades cognitivas: atención, memoria, fluidez verbal, lenguaje y habilidades visoespaciales. El puntaje de ACE se obtendrá para cada sujeto al inicio del estudio (antes de la intervención) y después de la intervención.

3.9. Resultados del Estudio

- **Resultado Primario**
    - Mejora global de las habilidades cognitivas.
- **Resultados Secundarios - Relacionados con las Habilidades Cognitivas**
    - Mejora de la habilidad de memoria.
    - Mejora de la habilidad de atención.
    - Mejora de la habilidad de orientación.
    - Mejora de la habilidad de fluidez verbal.
    - Mejora de la habilidad de lenguaje.
    - Mejora de las habilidades visuoespaciales.

---

4. Definiciones de los Resultados

4.1. Resultado Primario

El resultado primario del estudio, mejora de las habilidades cognitivas, será evaluado mediante la diferencia entre el puntaje de ACE posterior a la intervención y el puntaje de ACE previo a la intervención. Este puntaje tiene un rango de 0 a 100.

4.2. Resultados Secundarios

- **Resultados relacionados con las habilidades cognitivas**
  El puntaje de ACE comprende seis dimensiones, cada una evaluando un dominio

diferente: Memoria, atención, orientación, fluidez verbal, lenguaje y habilidades visoespaciales.
Cada mejora en las habilidades será evaluada utilizando las respectivas subdimensiones del puntaje ACE y computando la diferencia entre el subpuntaje post intervención y el subpuntaje pre intervención:

- Mejora en el subpuntaje de la habilidad de memoria.
- Mejora en el subpuntaje de la habilidad de atención.
- Mejora en el subpuntaje de la habilidad de orientación.
- Mejora en el subpuntaje de la habilidad de fluidez verbal.
- Mejora en el subpuntaje de la habilidad de lenguaje.
- Mejora en el subpuntaje de la habilidad de habilidades visoespaciales.

El subpuntaje de memoria varía entre 0 y 26, el subpuntaje de atención entre 0 y 8, el subpuntaje de orientación entre 0 y 10, el subpuntaje de fluidez verbal entre 0 y 14, el subpuntaje de lenguaje entre 0 y 16, y el subpuntaje de habilidades visoespaciales entre 0 y 26.

- **Resultados relacionados con la adherencia**
  La adherencia al protocolo del estudio se evaluará mediante: (1) el número de días en que el participante completó ("días presenciales").

---

5. Análisis Estadísticos

5.1. Disposición de los Sujetos

Se utilizará un diagrama de flujo CONSORT para resumir el número de sujetos que fueron:

- Inscritpos.
- Asignados a cada grupo.
- Descontinuados en cada grupo.
- Incluidos en el análisis cognitivo en cada grupo.

5.2. Distribución de las Características Socio-Demográficas y Clínicas

Todas las variables continuas se resumirán utilizando las siguientes estadísticas descriptivas: media, desviación estándar (SD) y rango, si los datos presentan una distribución normal, y mediana, rango intercuartil (IQR) y rango, si los datos están sesgados. La frecuencia y los porcentajes (basados en el tamaño de muestra sin valores faltantes) de los niveles observados se reportarán para todas las medidas categóricas.

5.3. Métodos de Análisis Estadísticos

5.3.1. Objetivo 1

Se reportará el score medio obtenido y la desviación estándar (SD) del ACE basal para los grupos de intervención y control. Además, se presentará la mejora del puntaje desde el inicio hasta el final del programa para cada grupo.
Para evaluar el impacto de MTT24.5 sobre las habilidades cognitivas, se utilizará un modelo lineal para cada dominio cognitivo (global, memoria, atención y orientación, fluidez verbal, lenguaje, y habilidades visoespaciales). Cada modelo utilizará el valor de mejora del puntaje como variable de resultado y el grupo (intervención y control) como variable independiente. El término del grupo evaluará el efecto no estandarizado de la intervención, indicando si la mejora media en los puntajes (cambio desde la línea de base) difiere entre los grupos de control e intervención. El tamaño del efecto se define como la diferencia entre la mejora media en el grupo de intervención y la mejora media en el grupo de control.
Para calcular los tamaños de efecto estandarizados, se utilizará un modelo lineal, incorporando el resultado escalado para cada dominio. El resultado escalado se calculará utilizando la fórmula: (puntaje de mejora - media (puntaje de mejora) / SD(puntaje de mejora)). Esta transformación facilita las comparaciones entre diferentes puntajes.

Se evaluará el cumplimiento de las suposiciones del modelo, y si no se cumplen, se propondrán mejoras.

5.3.2. Objetivo 2

Para identificar posibles fenotipos de respuesta basados en características basales individuales (incluyendo edad, sexo, historial médico, medicamentos, historial familiar de demencia y puntaje basal de reserva cognitiva), se empleará un modelo lineal para cada covariable de interés. El resultado primario se modelará con la covariable de interés, la variable de grupo y su interacción. Este término de interacción evaluará si el efecto de la intervención varía según los grupos de covariables.

Se generará un gráfico de bosque mostrando el tamaño del efecto estandarizado para cada grupo de covariables.

Covariables/Subgrupos de interés:

- Sexo (femenino, masculino)
- Edad (≤65 años, >65 años)
- Años de Educación (≤12 años, >12 años)
- Reserva cognitiva (baja, alta)
- Puntaje ACE en la línea base (<85, >85)
- Historia de diabetes (sí, no)
- Historia de hipertensión (sí, no)
- Historia de dislipidemia (sí, no)
- Historia familiar de demencia (sí, no)
- Uso de Estatinas / AAS (sí, no)

5.4. Manejo de Datos Perdidos, No Usados y Espurios

Las variables con más del 20% de datos faltantes no serán incluidas en el análisis. Para las variables con menos del 20% de valores faltantes, primero se investigará y tratará de completar los datos faltantes. Los datos disponibles serán incluidos en los listados y tabulaciones de datos, mostrando para cada informe el total real. No se utilizarán técnicas de imputación para los datos faltantes.

5.5. Intervalos de Confianza y Valores p

Todos los tests estadísticos aplicables serán de dos colas. Los intervalos de confianza se calcularán al 95% para todos los tamaños de efecto y las estimaciones del modelo.

5.6. Software Estadístico Empleado

El análisis será realizado con el software estadístico R o SPSS.

5.7. Convenciones de Reporte

Los resultados se presentarán con sus respectivos intervalos de confianza (IC) al 95% y valores p para pruebas de significancia.

6. Tablas de Referencia

Figura 1: Diagrama CONSORT

Diagrama de flujo del CONSORT: Mostrará los pasos del proceso de selección de sujetos, asignación, análisis y resultado.

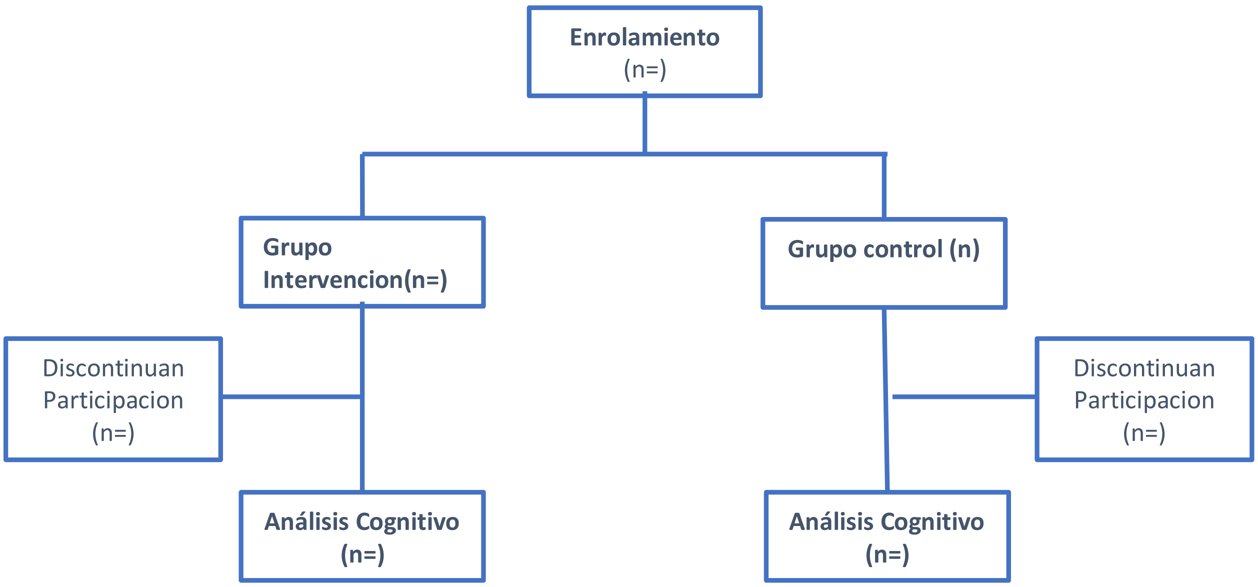

Tabla 1. Características Socio-Demográficas por Grupo

	Grupo Control	Grupo Intervencion
	n/N (%)	n/N (%)
Caracteristicas Socio-demograficas		
Sexo		
Masculino		
Femenino		
Edad*		
Edad categorizada		
<=65		
>65		
Años de educación *		
Dieta Mediterranea	-	
Actividad física	-	
Caracteristicas clinicas		
Reserva cognitiva reserve		
Baja		
Alta		
Diabetes		
Hipertension		
Dislipidemia		
Infarto de miocardio		
Accidente cerebrovascular		
Sme de apnea de sueño		
Historia familiar de demencia		

Tabla 2. Efecto del Programa MTT24.5 sobre los Resultados de las Habilidades Cognitivas

	**Grupo Control**	**Grupo Intervencion**	**Valor -P**
	**(N=)**	**(N=)**	
**Resultado primario**			
Habilidad cognitiva global			
Score basal, media (±DS)			
Cambio desde basal, media(DS)			
Efecto no-estandarizado (95% IC)*			
Efecto estandarizado (95% IC)**			
**Resultados secundarios**			
Atención			
Score basal, media (±DS)			
Cambio desde basal, media(DS)			
Efecto no-estandarizado (95% IC)*			
Efecto estandarizado (95% IC)**			
Orientacion			
Score basal, media (±DS)			
Cambio desde basal, media(DS)			
Efecto no-estandarizado (95% IC)*			
Efecto estandarizado (95% IC)**			
Memoria			

Score basal, media (±DS)<br>Cambio desde basal, media(DS)<br>Efecto no-estandarizado (95% IC)*<br>Efecto estandarizado (95% IC)**
Orientacion<br>Score basal, media (±DS)<br>Cambio desde basal, media(DS)<br>Efecto no-estandarizado (95% IC)*<br>Efecto estandarizado (95% IC)**
Lenguaje<br>Score basal, media (±DS)<br>Cambio desde basal, media(DS)<br>Efecto no-estandarizado (95% IC)*<br>Efecto estandarizado (95% IC)**
Habilidades visuoespaciales<br>Score basal, media (±DS)<br>Cambio desde basal, media(DS)<br>Efecto no-estandarizado (95% IC)*<br>Efecto estandarizado (95% IC)**

Abreviaturas: CI, intervalo de confianza; DS, desviación estándar
**Tamaño del efecto no estandarizado definido como la diferencia entre la mejora media en el grupo de intervención y la mejora media en el grupo de control.
**Tamaño del efecto estandarizado definido como la diferencia entre la media de la mejora escalada en el grupo de intervención y la media de la mejora escalada en el grupo de control.

Figura 2. Efecto del programa MTT 24.5 sobre las habilidades cognitivas según los grupos de covariables.
Se realizará diagrama de *Forest Plot*

---

7. Referencias

1. Ball K, Berch DB, Helmers KF, et al. Effects of Cognitive Training Interventions With Older Adults: A Randomized Controlled Trial. *JAMA.* 2002;288(18):2271–2281. doi:10.1001/jama.288.18.2271
2. Belleville S, Mellah S, Boller B, Ouellet É. Activation changes induced by cognitive training are consistent with improved cognitive reserve in older adults with subjective cognitive decline. Neurobiol Aging. 2023 Jan;121:107-118. doi: 10.1016/j.neurobiolaging.2022.10.010. Epub 2022 Oct 23. PMID: 36401900.
3. Megan A. Jennings, Robert A. Cribbie. Comparing Pre-Post Change Across Groups: Guidelines for Choosing between Difference Scores, ANCOVA, and Residual Change Scores, J. data sci. 14(2022), no. 2, 205-230, DOI 10.6339/JDS.201604_14(2).0002
4. Rebok GW, Ball K, Guey LT, Jones RN, Kim HY, King JW, Marsiske M, Morris JN, Tennstedt SL, Unverzagt FW, Willis SL; ACTIVE Study Group. Ten-year effects of the advanced cognitive training for independent and vital elderly cognitive training trial on cognition and everyday functioning in older adults. J Am Geriatr Soc. 2014 Jan;62(1):16-24. doi: 10.1111/jgs.12607. Epub 2014 Jan 13. PMID: 24417410; PMCID: PMC4055506.

5. Hardy JL, Nelson RA, Thomason ME, Sternberg DA, Katovich K, Farzin F, et al. (2015) Enhancing Cognitive Abilities with Comprehensive Training: A Large, Online, Randomized, Active- Controlled Trial. PLoS ONE 10(9): e0134467. doi:10.1371/journal.pone.0134467
6. Scarmeas N, Stern Y. Cognitive reserve and lifestyle. *J Clin Exp Neuropsychol*. 2003;25(5):625 633.
7. May A. experience-dependent structural plasticity in the adult human brain. *Trends Cogn Sci.* 2011; 15:475-82. doi: 10.1016/j. tics.2011.08.002
