# Supplementary material for: A new program for systematically enhancing cognitive reserve in healthy adults: A pilot randomized active-controlled clinical trial
Source: PLoS One. 2025 Oct 1;20(10):e0331193. doi: 10.1371/journal.pone.0331193 (PMC12488004; doi:10.1371/journal.pone.0331193)
Supplement: S4 File — (PDF) [file pone.0331193.s004.pdf]

ABBREVIATIONS AND CODE REFERENCES FOR LECTURE OF S1 DATA SET

ABBREVIATION	CONCEPT	CODE
SEX	SEX	FEMALE=0<br>MALE=1
AGE	AGE	n (numerical variable)
ED	YEARS OF EDUCATION	n (numerical variable)
CR	COGNITIVE RESERVE	LOW=0<br>HIGH=1
DBT	DIABETES	NO=0<br>YES=1
HYP	HYPERTENSION	NO=0<br>YES=1
DYSL	DYSLIPEMIA	NO=0<br>YES=1
MI	MYOCARDIAL INFARTION	NO=0<br>YES=1
ST	STROKE	NO=0<br>YES=1
OSA	OBSTRUCTIVE SLEEP APNEA SYNDROME	NO=0<br>YES=1
PARK	PARKINSON DISEASE	NO=0<br>YES=1
DA	DEMENTIA ANCESTORS	NO=0<br>YES=1
CMED	CHRONIC MEDICATION	NO=0<br>YES=1
STAT	STATINS	NO=0<br>YES=1
EZE	EZETIMIBE	NO=0<br>YES=1
HYPOGL	HYPOGLICEMIC DRUGS	NO=0<br>YES=1

ACE/ARA	ACE INHIBITORS/AGII RECEPTOR ANTAGONIST	NO=0<br>YES=1
BB	BETA BLOCKERS	NO=0<br>YES=1
CA	CALCIUM CHANNEL ANTAGONIST	NO=0<br>YES=1
AAS	ACETYLSALICYLIC ACID	NO=0<br>YES=1
TH	THYROID HORMONE	NO=0<br>YES=1
BZD	BENZODIACEPINES	NO=0<br>YES=1
O3	OMEGA 3	NO=0<br>YES=1
VITD	D VITAMIN	NO=0<br>YES=1
MEL	MELATONIN	NO=0<br>YES=1
MAG	MAGNESIUM	YES=1
ORIENT	ORIENTATION	0 A 8
ATTEN	ATTENTION	0 A 8
MEM	MEMORY	0 A 26
VFLUENCY	VERBAL FLUENCY	0 A 14
VISUOES	VISUOSPATIAL SKILLS	0 A 26
ACE	ADDENBROOK'S COGNITIVE TEST (ACE) BASELINE	0-100
