# Supplementary material for: A new program for systematically enhancing cognitive reserve in healthy adults: A pilot randomized active-controlled clinical trial
Source: PLoS One. 2025 Oct 1;20(10):e0331193. doi: 10.1371/journal.pone.0331193 (PMC12488004; doi:10.1371/journal.pone.0331193)
Supplement: Attachment — Submitted filename: RESPONSE TO REVIEWERS .pdf [file pone.0331193.s006.pdf]

April 7, 2025.

Response letter to Academic Editor and to Reviewers

PONE-D-24-57989: A New Program for Systematically Enhancing Cognitive Reserve in Healthy Adults: A Pilot Randomized Active-Controlled Study

Dear Dr. Khanmohammadi, Ph.D, Academic Editor

Dear Reviewers, #1 and #2,

We would like to sincerely thank you for the time and effort you have dedicated to reviewing our manuscript. We deeply appreciate your thoughtful comments and feedback. We are very hopeful to communicate our findings to our colleagues through the prestigious scientific journal, PLOS ONE.

Below, we respond to each of the suggestions, comments, and recommendations from the reviewers. We also confirm that we have made the necessary changes and additions to meet all the requirements outlined in your evaluation letter.

We believe these changes address all the concerns raised and have significantly improved the manuscript. We are grateful for your guidance throughout this process and look forward to your feedback.

Thank you once again for your time and support.

Sincerely,

Carol Kotliar, PhD

I. Actions and changes made in response to editorial requirement

- We ensured that the manuscript meets PLOS ONE's style requirements, including those for file naming, and followed the recommended template provided in the formatting sample link.
- The Inclusivity Form has been included in the supplementary material.
- As requested by Reviewer #2, all data underlying the findings described in our manuscript are now freely available to other researchers, with the data rows included in the supplementary material.
- The title on the online submission form and the title in the manuscript are identical, as you instructed us to do.
- We have included the abstract after the title page as per your recommendation.
- Captions for the Supporting Information files have been added at the end of the manuscript, and we have updated all in-text citations to match accordingly.

II. Responses to Reviewer #1

Section: Introduction

1. Comment: *"Throughout the entire introduction, the authors include statements, commentary, and previous studies without citations. Given that the reference list includes more citations than what was used in this section, perhaps some were left out."*

Response: Following your observation, we have included the appropriate citations for all statements, commentary, and previous studies as requested. The reference list has been reviewed and updated to complement this valuable observation. ***(#See pages 3 to 5 for the updated introduction with the corresponding citations from references 1 to 28, which can be found in the references section on pages 24 to 27)***

2. Comment: *"Rather than broadly discussing how learning interventions improve 'neuroplasticity' and 'cognitive function,' address how these interventions have improved the cognitive functions measured in this study, such as memory, attention, verbal fluency, language, and visuospatial skills. Providing this extra clarity can strengthen the introduction."*

Response: We consider your comment extremely valuable in strengthening the focus of the introduction on our study's specific scope. We have restructured and enhanced the introduction, providing a foundation with the corresponding citations that address how learning interventions have improved the specific functions measured in this study. ***(#See the modifications on pages 3 to 5 throughout the entire revised introduction)***

3. **Comment:** *"A stronger focus on studies involving healthy older adults would better support the study's rationale and help formulate a more specific hypothesis. The authors state that this pilot study aims to develop a hypothesis to validate the effectiveness of the MTT24.5 for improving cognitive performance, however, this approach may hinder the validity of this technique as a more precise hypothesis could have been formulated based on the previous success of cognitive learning interventions with similar approaches in healthy older adults."*

Response: We also appreciate this valuable comment. We have now focused more on studies involving healthy adults in the introduction and emphasized this condition among of the adults included in the study in the formulation of the hypothesis. Additionally, we thank the reviewer for highlighting this specific characteristic of our population, which could undoubtedly be considered for future prevention studies to be applied in the general population. Given the increased life expectancy, brain stimulation or training could be a potential recommendation to contribute to the reduction of future cognitive decline risk. ***(See the modifications on pages 3 to 5 throughout the entire revised introduction)***

Section: Method

4. **Comment:** *"While the pairs (DATA and TECHS) are introduced and methodologically explained thoroughly, it remains theoretically unclear why the authors chose these pairs and how they both contributed to the study's objective."*

Response: Thank you for your valuable question. The origin of the MTT design, utilizing binomial units of "DATA" and "TECHS," was proposed by the lead author of the manuscript, Carol Kotliar, PhD, who also registered it as an intellectual property. The core idea was to create a structured, progressive learning system that would introduce new knowledge over time. The term "DATA" was chosen to describe these new units of knowledge, each represented as a simple statement encapsulated in a concise sentence. The original concept was to design the knowledge in such a way that each "DATA" unit could stand independently without requiring interconnection with others. To achieve this, the knowledge was carefully selected from four main scientific fields: cultural, formal (including logic and mathematics), biological, and social sciences. This diversified approach aimed to stimulate various areas of the brain, with some of the knowledge deliberately positioned outside the participant's usual domain of expertise. Given that it is unlikely an individual would have a professional or personal interest in all these areas, this strategy ensured broader cognitive engagement, pushing participants beyond their comfort zones and established knowledge base.

Data for each unit was carefully sourced using search engines focusing on these four fields. Each "DATA" point was distilled into a single, easily memorable sentence. To enhance the engagement and accessibility of the material,

additional resources such as videos, images, anecdotes, and emotional cues were incorporated alongside the core content.

In parallel, the term "TECHS" was coined to describe the methodologies used to facilitate the learning of the "DATA." These techniques were selected based on their documented association with neuroplastic changes in the brain, as evidenced by structural and functional studies in the scientific literature. Among the various techniques explored, Braille literacy was identified as particularly effective, with approximately 60% of the "TECHS" incorporating Braille reading and writing exercises. This inclusion was grounded in the evidence of its neuroplastic benefits, stimulating brain regions associated with sensory integration, motor control, and memory retention.

Participants in the MTT program developed Braille reading and writing skills as part of the curriculum, as well as the ability to perform mirrored writing with both hands simultaneously. The content written and read always corresponded to the new "DATA," reinforcing the memorization process throughout the activities.

The dual structure of the MTT program comprising "DATA" (the new knowledge) and "TECHS" (the methods for learning) was designed to promote dynamic cognitive engagement. By offering a varied and structured approach, it aimed to enhance neuroplasticity through the stimulation of multiple brain regions and encourage the development of novel cognitive pathways in healthy adults. ***(A summarized expansion of this justification for the Data-Tech pair has been included in the Methods section on pages 11 and 12)***

5. **Comment:** *"A few minor methodological details are missing in this design. For example, how were these pairs administered, executed, and monitored? Were the pairs performed digitally or in a classroom setting?"*

Response: The binomial Data-Tech pair is presented in a classroom setting. The sessions are taught in person by a certified trainer who uses standardized materials, including videos, images, oral descriptions, and various resources such as sheets of paper, pencils, Braille tablets with punchers, a luminous button, a semi-baked ball for foot pressure, a calculator, Wisconsin cards, the Stroop Color Test, and other tools integrated into the techniques. Each participant is assigned specific materials. There is one trainer for every 20 participants, with an assistant; the number of assistants increases as the class size grows. To complete the full MTT, participants must attend 12 consecutive sessions.

The program is executed sequentially, with all data sets organized for each class. Each session combines one piece of knowledge from each of the four scientific fields cultural, social, formal, and biological sciences; administered in a specific order across the 12 sessions, along with their corresponding techniques. The trainer explains each piece of data to all participants during each session, followed by the techniques associated with that data. After completing the first data-tech pair, the process progresses to the second, then the third, continuing in this structured manner throughout each session.

A participant is considered to have completed the program if they attend at least 80% of the sessions. If a participant is unable to attend a session, they are encouraged to make it up in a later class, but they cannot miss any classes without attending a make-up session. ***(#Following your recommendation, the description is expanded in the Methods section on page 10.)"***

III. Responses to reviewer #2

Section: Introduction

1. **Comment:** *"Providing a deeper explanation of how MTT24.5 promotes neuroplasticity would enhance the manuscript. Discussing theoretical frameworks, such as network control theory, could offer valuable insights into the underlying mechanisms."*

   Response: To address the reviewer's request, we provided a deeper explanation of how the **Mental Training Tech 24.5 (MTT24.5)** program may promote neuroplasticity by integrating the theoretical framework of **Network Control Theory (NCT)**. This analysis will link the effects of the program on cognitive functions (attention, memory, verbal fluency, visuospatial skills, and processing speed) to the principles of NCT. We appreciate this valuable suggestion, which has been incorporated into the manuscript. Considering that this perspective adds depth to the interpretation of the results, we believe that its inclusion in the discussion strongly enhances the impact of these concepts.

   We believe this significantly enriches our manuscript. *(#See the pages 17 and 18)*

2. **Comment:** *"The last sentence of the introduction states that 'This cohort represents the general population.' Is this study a clinical trial or a cohort study?"*

   Response: This was certainly necessary to modify. We have updated the last sentence of the introduction, clarifying that the study is a clinical trial, not a cohort study. *(#See the correction in introduction section at page 5)*

Section: Method

3. **Comment:** *"Why is the ratio of the intervention group to the control group considered to be 2:1?"*

   Response: A 2:1 randomization ratio was chosen to facilitate recruitment among healthy volunteers, offering a higher chance of receiving the Intervention. In the case of potentially improving cognitive function, this ratio,

which allocates more 'healthy' participants to the experimental treatment in a short period, can promote better adherence and recruitment. This approach was particularly relevant given that this was a pilot study. By using this design, a larger sample size in the intervention group was achieved. The decision was made after careful consideration of study resources and ethical guidelines to ensure a fair and scientifically sound comparison between groups.

Section: Results

4. Comment (d) of Reviewer #2: *"The results section of the paper includes the demographic information of the participants. However, this table would be more appropriate if presented in the Methods section*."

4.1 Response: The table describing the demographic information of the participants has been moved to the Methods section as suggested. ***(#see page 9)***
