# Supplementary material for: A new program for systematically enhancing cognitive reserve in healthy adults: A pilot randomized active-controlled clinical trial
Source: PLoS One. 2025 Oct 1;20(10):e0331193. doi: 10.1371/journal.pone.0331193 (PMC12488004; doi:10.1371/journal.pone.0331193)
Supplement: Attachment — Submitted filename: RESPONSE TO REVIEWERS (2).docx [file pone.0331193.s007.docx]

May 18, 2025.

***Response letter to Academic Editor and to Reviewers***

**PONE-D-24-57989: A New Program for Systematically Enhancing Cognitive Reserve in Healthy Adults: A Pilot Randomized Active-Controlled Study**

*Dear Dr. Khanmohammadi, Ph.D, Academic Editor*

*Dear Reviewers, #1 and #2*

We would like to express our sincere gratitude for the time and effort you have dedicated to reviewing our manuscript. We greatly appreciate your thoughtful feedback. In response to your comments, we have made the necessary revisions to the manuscript, as detailed below.

The two changes requested by Reviewer #1 were implemented, including the mention of the study on the cognitive platform Lumosity in the Introduction, and a rewritten description of the intervention in the Methods section to enhance clarity. Due to the inclusion of the Hardy et al. study in the Introduction, its position in the reference list was updated, which required adjusting the overall numbering of references accordingly.

These revisions address the points raised and we believe they have further strengthened the manuscript. We are hopeful that this second revised version meets your expectations and look forward to your feedback.

Thank you once again for your valuable support.

Sincerely,
Carol Kotliar, PhD

**Responses to Reviewer #1**

**1.	Comment (*Introduction*)**: *“At the end of the discussion, the authors mention that their findings are consistent with online cognitive training platforms such as Lumosity. However, this is the first time the authors mention this online cognitive training platform, only at the end of the paper. Considering both Luminosity and the current study highlight similar benefits of enhanced cognitive abilities, it would strengthen the introduction and display novelty to the current study if Lumosity were introduced before the current study.”*

**Response:** Thank you for your valuable observation regarding the mention of Lumosity. We agree that introducing this online cognitive training platform earlier in the manuscript improves the context and helps emphasize the novelty of our study. Following your recommendation, we have now included a mention of Lumosity in the Introduction section (page 3, lines 63 to 69).

2.	**Comment (*Methods****):” The authors provided information on how the DATA and TECHS components are used for the intervention; however, the overall organization of these concepts throughout the methods section is not optimal. Especially when initially introducing the components, they are abstract and contain numerous redundancies. As these two components are central to the intervention, it is crucial that they are explained more clearly.”*

**Response:** We appreciate your comment regarding the organization and clarity of the DATA and TECHS components in the Methods section. We understand the importance of providing a clear and concise explanation, especially as these two components are central to the intervention. In response to your requirement, we have revised the description to improve clarity, eliminating abstract terms and redundant sentences. We have carefully reviewed the revised text to ensure its clarity and coherence. Please refer to pages 9 to 11 for the updated description.
