# Supplementary material for: A new program for systematically enhancing cognitive reserve in healthy adults: A pilot randomized active-controlled clinical trial
Source: PLoS One. 2025 Oct 1;20(10):e0331193. doi: 10.1371/journal.pone.0331193 (PMC12488004; doi:10.1371/journal.pone.0331193)
Supplement: Attachment — Submitted filename: Response to Reviewer #3.docx [file pone.0331193.s009.docx]

Agost 1, 2025.

PONE-D-24-57989R3

A New Program for Systematically Enhancing Cognitive Reserve in Healthy Adults: A Pilot Randomized Active-Controlled Trial

PLOS ONE

***Response letter to Academic Editor and to Reviewer #3***

*Dear Reviewer, #3*

In response to your comments, we have made the necessary revisions to the manuscript, as detailed below.

The 3 minor revisions requested were implemented, including their detailed description for the readers inside the manuscript.

These revisions address the points raised and we believe they have further strengthened the manuscript. We are hopeful that this third revised version meets your expectations and look forward to the final acceptance of the manuscript.

Thank you once again for your valuable support.

Sincerely,
Carol Kotliar, PhD

**Responses to Reviewer #3**

**1.	Comment:** *“***Sample size justification** – **Please clarify the rationale for using an effect size of 0.80. Was this based on prior literature, previous data, or another source?**

**Response**: The effect size of 0.80 used in our sample size calculation was based on prior literature, particularly the methodology and effect size interpretation used in the ACTIVE trial (Advanced Cognitive Training for Independent and Vital Elderly).

Although the observed effects in ACTIVE ranged from small to moderate (e.g., 0.23 for reasoning and 0.66 for speed), we considered an effect size of 0.80 as a conservative target to detect a meaningful difference with sufficient power, assuming a best-case scenario for the intervention's impact.

Additionally, we considered the characteristics of our primary cognitive outcome measure. For example, in the Addenbrooke’s Cognitive Examination (ACE), a 1-point difference is clinically relevant for identifying cognitive decline. Therefore, assuming an intervention could achieve at least a moderate to large improvement (corresponding to 4 points, SD = 5.4, d = 0.8) was both clinically reasonable and consistent with previous literature on cognitive training effects.

*We introduced this explanation also in the manuscript in the corresponding section to inform the reader. (Page 13)*

2. **Comment:** “**Multiple comparisons”** **Table 2 includes several secondary cognitive outcomes. Please indicate whether any corrections for multiple comparisons were applied, particularly for measures such as attention, verbal fluency, and visuospatial skills. Even in a pilot study, this should be addressed.**

**Response:** Thank you for this important comment. We fully agree that the issue of multiple comparisons should be addressed, even in a pilot study. Given the exploratory nature of this pilot study, no corrections for multiple comparisons were applied. This decision was made to maximize sensitivity to potential signals that could inform future confirmatory studies. In pilot or exploratory studies like this one, it’s common not to apply these corrections. In these kinds of studies, the focus is more on detecting potential signals of an effect, even if that means accepting a higher risk of false positives.

Following your suggestion, we addressed it with a clearly state of this approach in the methods section as follows:

**Changes in the manuscript:**

A) We have added the following sentence to the Statistical Analysis section (PAGE 13): *"* ***Given the exploratory nature of this pilot study, no corrections for multiple comparisons were applied. This approach was chosen to prioritize sensitivity to potential signals that could inform future confirmatory research.”***

*B)* We have also added a corresponding footnote in the manuscript at Table 2 (PAGE 14)

**"P-values are unadjusted for multiple comparisons due to the exploratory nature of the study."**

**3. Comment: “Randomization ratio** – **While the 2:1 randomization appears justifiable, kindly explain the rationale for this choice explicitly in the manuscript to inform the reader.”**

**Response**: A 2:1 randomization ratio was choice to support recruitment and enhance adherence among healthy volunteers by increasing the likelihood of being assigned to the intervention group. In studies involving cognitive enhancement in healthy populations, such an approach can be particularly effective in motivating participation. Given the nature of this pilot study, a larger sample size in the intervention arm allowed for a more informative preliminary assessment of feasibility and potential effects. This decision was made after careful consideration of ethical principles, study objectives, and available resources, ensuring a scientifically valid comparison between groups.

***# See at manuscript PAGE 6: We incorporated the explanation as you requested into the manuscript to inform the reader.***

***Best regards,***

***Carol Kotliar, PhD***

.
